# Equine Myxovirus Resistance Protein 2 Restricts Lentiviral Replication by Blocking Nuclear Uptake of Capsid Protein

**DOI:** 10.1128/JVI.00499-18

**Published:** 2018-08-29

**Authors:** Shuang Ji, Lei Na, Huiling Ren, Yujie Wang, Xiaojun Wang

**Affiliations:** a State Key Laboratory of Veterinary Biotechnology, Harbin Veterinary Research Institute, Chinese Academy of Agricultural Sciences, Harbin, China; Icahn School of Medicine at Mount Sinai

**Keywords:** HIV-1, interferon, Mx2, antiviral activity, capsid binding, equine infectious anemia virus, lentiviruses, position change

## Abstract

Human myxovirus resistance protein 2 (huMxB) has been shown to be a determinant type I interferon (IFN)-induced host factor involved in the inhibition of human immunodeficiency virus type 1 (HIV-1) as well as many other primate lentiviruses. This blocking occurs after the reverse transcription of viral RNA and ahead of integration into the host DNA, which is closely connected to the ability of the protein to bind the viral capsid. To date, Mx2s derived from nonprimate animals have shown no capacity for HIV-1 suppression. In this study, we examined the restrictive effect of equine Mx2 (eqMx2) on both equine infectious anemia virus (EIAV) and HIV-1 and investigated possible mechanisms for its specific function. We demonstrated that IFN-α/β upregulates the expression of eqMx2 in equine monocyte-derived macrophages (eMDMs). The overexpression of eqMx2 significantly suppresses the replication of EIAV, HIV-1, and simian immunodeficiency viruses (SIVs) but not that of murine leukemia virus (MLV). The knockdown of eqMx2 transcription weakens the inhibition of EIAV replication by type I interferon. Interestingly, data from immunofluorescence assays suggest that the subcellular localization of eqMx2 changes following virus infection, from being dispersed in the cytoplasm to being accumulated at the nuclear envelope. Furthermore, eqMx2 blocks the nuclear uptake of the proviral genome by binding to the viral capsid. The N-terminally truncated mutant of eqMx2 lost the ability to bind the viral capsid as well as the restriction effect for lentiviruses. These results improve our understanding of the Mx2 protein in nonprimate animals.

**IMPORTANCE** Previous research has shown that the antiviral ability of Mx2s is confined to primates, particularly humans. EIAV has been shown to be insensitive to restriction by human MxB. Here, we describe the function of equine Mx2. This protein plays an important role in the suppression of EIAV, HIV-1, and SIVs. The antiviral activity of eqMx2 depends on its subcellular location as well as its capsid binding capacity. Our results showed that following viral infection, eqMx2 changes its original cytoplasmic location and accumulates at the nuclear envelope, where it binds to the viral capsid and blocks the nuclear entry of reverse-transcribed proviral DNAs. In contrast, huMxB does not bind to the EIAV capsid and shows no EIAV restriction effect. These studies expand our understanding of the function of the equine Mx2 protein.

## Abstract (precis)

Previous research has shown that the antiviral ability of Mx2s is confined to primates, particularly humans. EIAV has been shown to be insensitive to restriction by human MxB. Here, we describe the function of equine Mx2. This protein plays an important role in the suppression of EIAV, HIV-1, and SIVs. The antiviral activity of eqMx2 depends on its subcellular location as well as its capsid binding capacity. Our results showed that following viral infection, eqMx2 changes its original cytoplasmic location and accumulates at the nuclear envelope, where it binds to the viral capsid and blocks the nuclear entry of reverse-transcribed proviral DNAs. In contrast, huMxB does not bind to the EIAV capsid and shows no EIAV restriction effect. These studies expand our understanding of the function of the equine Mx2 protein.

## INTRODUCTION

Myxovirus resistance proteins (Mxs) are interferon (IFN)-inducible intracellular restriction effectors that belong to the dynamin-like GTPase family and have a broad range of antiviral functions (1, 2). In humans, the two members of the Mx family, MxA and MxB, are encoded by closely connected genes on chromosome 21 but have different antiviral profiles (1, 3). The MxA protein has long been recognized as a marker gene for interferon action and suppresses a wide range of DNA and RNA viruses, including influenza (4, 5), measles (6), Hantaan (7), and La Crosse (8) viruses as well as many other pathogenic viruses (2). MxB, however, was always thought to lack antiviral activity, instead contributing to certain basic cell functions (3), such as regulating nucleocytoplasmic transport (9) and cell cycle progression (10). The antiviral activity of MxB was first reported in 2013 (11–13), when it was shown that the human MxB (huMxB) protein plays a crucial role in blocking human immunodeficiency virus type 1 (HIV-1) infection in interferon-induced human cells.

Human MxB shares 63% amino acid sequence identity with MxA, so the overall structures of these proteins are similar. In both proteins, three bundle signaling element (BSE) domains are separated by a globular GTPase domain and a stalk domain (14). The functional protein is an extended antiparallel dimer formed of two MxB monomers (14, 15). The unstructured N-terminal and C-terminal domains of MxB are responsible for HIV-1 capsid (CA) binding and oligomerization, respectively, both of which are considered indispensable for its antiviral activity (14, 16–20). There are two isoforms of huMxB with different subcellular localizations: the long 78-kDa form located primarily at the nuclear envelope and the short 76-kDa form found mostly in the cytoplasm (21). The nuclear envelope localization of full-length MxB is thought to be crucial for blocking HIV-1 because both cytoplasmic human MxA and N-terminally truncated MxB lack this function (21–23). Moreover, appending the N-terminal 91 residues of huMxB to huMxA is sufficient to target it to the nuclear envelope and potently suppress HIV-1 (23). Nevertheless, some studies have suggested that the N terminus of full-length MxB contributes to the limitation of HIV-1 infection by binding the HIV-1 capsid with its ^11^RRR^13^ motif rather than acting as a nuclear localization signal (20, 24).

Mx2s from many other nonhuman primates have also been reported to inhibit infection with HIV-1, whereas Mx2s from other mammals (ovine and canine) lack this function (22). To date, no data claiming its antiviral activity in nonprimate Mx2 proteins have been reported. Meanwhile, human MxB shows little or no ability to limit infection by nonprimate lentiviruses, including equine infectious anemia virus (EIAV) (11, 12, 22). EIAV, a lentivirus similar to HIV-1 (25), can be inhibited by many host restriction factors (26, 27). It is unknown whether equine cells encode an orthologue of human MxB. Moreover, the interactions between equine Mx2 (eqMx2) and EIAV or other retroviruses also remain unknown. In this study, we isolated the equine Mx2 gene and investigated its antiviral activity. We found that, unlike those of other nonprimate animals, equine Mx2 can inhibit the replication of many lentiviruses, including HIV-1, and that this ability is closely associated with blocking the nuclear uptake of the viral capsid.

## RESULTS

### Induction of equine Mx2 mRNA expression by type I interferon.

**FIG 1:**
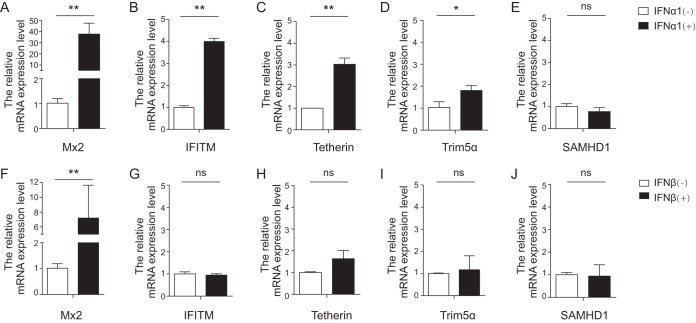
Induction of expression of multiple equine restriction factors by type I IFN. eMDMs were cultured for 24 h in the presence (+) or absence (−) of equine IFN-α1. (A to E) The transcription levels of equine Mx2 (A), IFITM (B), tetherin (C), Trim5α (D), SAMHD1 (E), and β-actin were quantified using real-time PCR. The numbers of mRNA copies were normalized to those of β-actin. (F to J) Same experimental procedure as for panels A to E, except that cells were treated with equine IFN-β. The data represent the means ± standard errors (SE) from three independent experiments (*, *P* < 0.05; **, *P* < 0.01; ns, no significance). *P* values of <0.05 were considered statistically significant.

To determine whether the expression of equine Mx2 could be induced by type I interferon, a relative real-time reverse transcriptase PCR (RT-PCR) analysis was performed. Equine monocyte-derived macrophages (eMDMs) from three horses were separately treated with either equine IFN-α1 or IFN-β for 24 h at 100 ng/μl. Relative mRNA expression levels were determined using the 2*^−ΔΔCT^* method. Twenty-four hours following exposure to IFN-α1 and IFN-β, the expression of equine Mx2 was significantly increased by averages of 37.8-fold and 7.2-fold, respectively (Fig. 1A and F). Some other well-studied host restriction factors, including equine IFITM, tetherin, Trim5α, and SAMHD1, were also examined. The expression of IFITM and tetherin could be upregulated by IFN-α1 3- to 4-fold (Fig. 1B and C), that of Trim5α could be upregulated about 2-fold (Fig. 1D), and there was almost no change for SAMHD1 (Fig. 1E). With the exception of eqMx2, there was no obvious induction of the restriction factors by IFN-β in 24 h (IFITM [Fig. 1G], tetherin [Fig. 1H], Trim5α [Fig. 1I], or SAMHD1 [Fig. 1J]). Taken together, these results show that the expression of eqMx2 is more strongly induced by type I interferon in 24 h than those of other equine restriction factors, suggesting that eqMx2 may play an important role in interferon-induced antiviral activity.

### Sequence comparison and analysis of equine Mx2.

**FIG 2:**
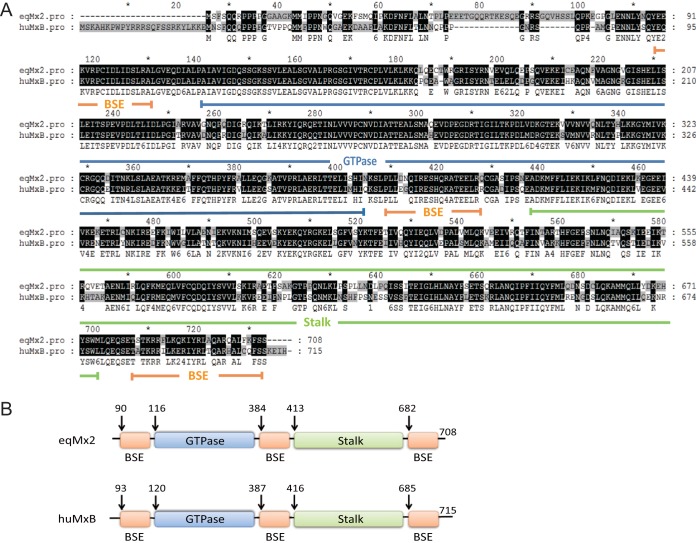
Cloning and sequence analysis of the equine Mx2 gene. (A) Cloning of eqMx2 cDNA. eqMx2 cDNA was cloned by RT-PCR from total RNA extracted from eMDMs previously treated with equine IFN-α1 (100 ng/μl). The deduced amino acid sequence was aligned with that of human MxB. Major functional domains, including the GTPase domain, stalk domain, and BSE domains, are marked with blue, green, and orange lines, respectively. (B) Schematic of human and predicted equine Mx2 structures, with residues of domain boundaries denoted and colored. The GTPase (blue) and stalk (green) domains are located at either end of equine Mx2, bridged by three BSE domains (orange). The colors correspond to the domains in panel A. The arrows in the schematic denote the first and last visible residues in the structure.

To investigate the function of equine Mx2, primers were designed according to the predicted sequence in GenBank (GenBank accession number XM_005606159.2) to clone the equine Mx2 gene. A 2,127-bp fragment was amplified by RT-PCR from eMDMs treated with IFN-α1 for 24 h. The cells were treated with interferon because the basic expression level of eqMx2 is quite low and hard to amplify. Using bioinformatics prediction software (SWISS-MODEL and Phyre2), equine Mx2 was predicted to be structurally similar to human MxB. The GTPase and stalk domains are located at either end of equine Mx2, bridged by three BSE domains (Fig. 2A and B). The equine Mx2 amino acid sequence shares 75.5% sequence identity with human MxB (GenBank accession number NM_002463.1). The greatest differences between these two proteins are in the unstructured N-terminal region. Interestingly, several papers report that the N terminus of human MxB is indispensable for its function to restrict HIV-1 (12, 17, 20, 23, 24), which prompted our investigation into the antiviral activity of equine Mx2.

### Equine Mx2 inhibits the replication of HIV-1 and reduces the entry of HIV-1 cDNA into the nucleus.

**FIG 3:**
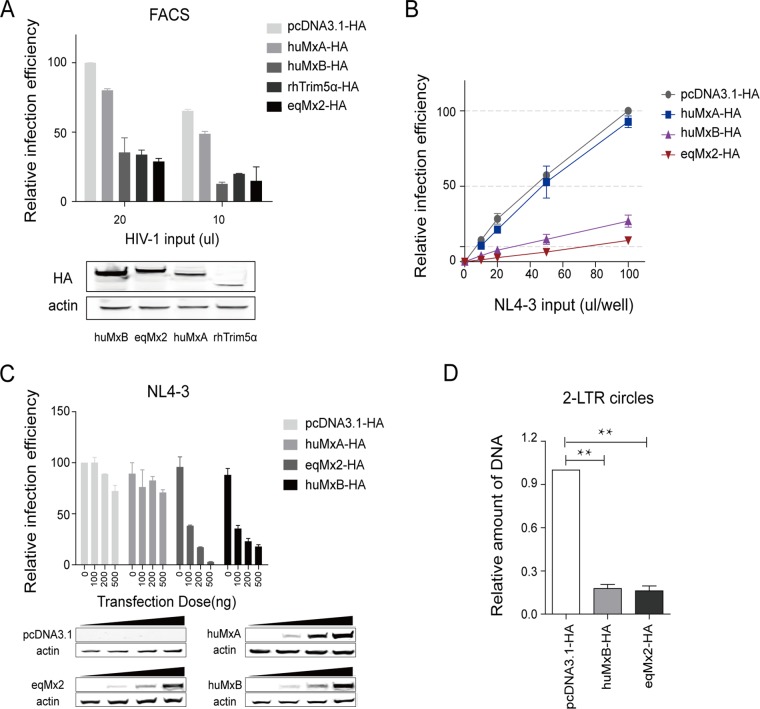
Equine Mx2 inhibits HIV-1 infection and nuclear uptake of HIV-1 cDNA. (A) HEK293T cells were transfected with 1 μg plasmids expressing either huMxA, huMxB, rhTRIM5α, or eqMx2. huMxB and rhTRIM5α served as positive controls for restricting the early life stages of HIV-1. An empty plasmid vector (pcDNA3.1-HA) and huMxA were used as the negative controls. Two doses of HIV-1 GFP reporter viruses were inoculated at 24 hpt. Cells were lysed, and the relative single-cycle infection efficiency in each cell lysate was calculated by flow cytometry at 24 hpi. This experiment was performed three times, and the mean results with standard deviations are shown. Intracellular expression of HA-tagged huMxB, eqMx2, huMxA, and rhTRIM5α was confirmed by Western blotting using an anti-HA antibody, and staining for actin served as a loading control. (B) HEK293T cells were transfected with 1 μg plasmids expressing either pcDNA3.1, huMxA (negative control), huMxB (positive control), or eqMx2 and inoculated with increasing amounts (0, 10, 20, 50, and 100 μl) of a one-life-cycle HIV-1 luciferase reporter virus (NL4-3). Cells were lysed, and the infection efficiency relative to pcDNA3.1-HA was monitored at 24 hpi. Mean relative infection efficiencies with standard deviations from three independent experiments are shown. (C) HEK293T cells were transfected with increasing amounts (0, 100, 200, and 500 ng) of huMxA, eqMx2, and huMxB expression plasmids and inoculated with HIV-1_NL4-3_ at 2 ng reverse transcriptase (RT) activity at 24 hpi. Cells were lysed, and luciferase activity in the cell lysates was measured at 24 hpi. The data represent the means ± SE from three independent experiments. Dosage-dependent expression of certain Mxs was identified by Western blotting using an anti-HA antibody, and actin served as a loading control. (D) Equine Mx2 inhibits the nuclear uptake of HIV-1 cDNA. HEK293T cells were transduced with an empty vector (pcDNA3.1-HA) or huMxB- or eqMx2-expressing plasmids and challenged with HIV-1_NL4-3_ pseudotyped virus at 24 hpt. The total DNA was collected from the cells 24 h after infection, and we carried out qPCR analysis of 2-LTR circular DNA, normalized using GAPDH. The results represent the means ± SE from three independent experiments (**, *P* < 0.01).

To investigate whether the equine Mx2 protein has the ability to restrict the replication of certain lentiviruses, and to compare its function with that of huMxB, we cloned these two genes into a pcDNA3.1(+) vector with two-hemagglutinin (2×HA) tags at the C terminus and expressed them in HEK293T cells. huMxA and rhesus macaque TRIM5α (rhTRIM5α) were also expressed as negative (1, 23) and positive (28–30) controls, respectively (Fig. 3A). HEK293T cells were transfected with either an empty vector with only the HA tag (pcDNA3.1-HA) or the expression vector for huMxA, huMxB, rhTRIM5α, or eqMx2. First, we used green fluorescent protein (GFP) reporter-pseudotyped HIV-1 to infect cells overexpressing certain proteins and calculated the relative single-cycle infection efficiency using flow cytometry. The results suggest that like huMxB and rhTrim5α, eqMx2 can also restrict infection by GFP reporter-pseudotyped HIV-1 (Fig. 3A). To further confirm the effect of eqMx2 on HIV-1 replication, a luciferase-expressing HIV-1_NL4-3_ reporter virus (NL4-3) was generated. HEK293T cells were transfected with either the expression vector for huMxA, huMxB, or eqMx2 or an empty vector control. The NL4-3 pseudotyped virus was inoculated at 24 h posttransfection (hpt). Luciferase activity was detected at 24 h postinfection (hpi) and demonstrated that the replication of HIV-1 could also be potently restricted by eqMx2, and the restrictive effect of eqMx2 was almost the same as that of huMxB (Fig. 3B). Increasing the doses of huMxB and eqMx2 expression proportionally reduced the infection efficiency of NL4-3, thus demonstrating that the inhibition of these proteins was dose dependent (Fig. 3C).

We further measured the amount of HIV-1 2-long-terminal-repeat (2-LTR) circular DNA, which is a well-known marker for the nuclear uptake of HIV-1 cDNA. HEK293T cells were transduced with a negative control (pcDNA3.1-HA) or the eqMx2 or huMxB expression vector and challenged with HIV-1_NL4-3_ pseudotyped virus. Total DNA was collected at 24 hpi, and the number of 2-LTR circles was assessed using quantitative PCR (qPCR). The glyceraldehyde-3-phosphate dehydrogenase (GAPDH) gene was used as a reference gene to normalize the number of living cells. The results suggested that the amount of HIV-1 2-LTR circular DNA can be reduced almost equally by eqMx2 and huMxB (Fig. 3D). In short, these data suggested that eqMx2 inhibits infection by HIV-1 by blocking the nuclear uptake of HIV-1 reverse transcription products.

### Equine Mx2 restricts EIAV replication and is required for type I IFN-induced EIAV suppression.

**FIG 4:**
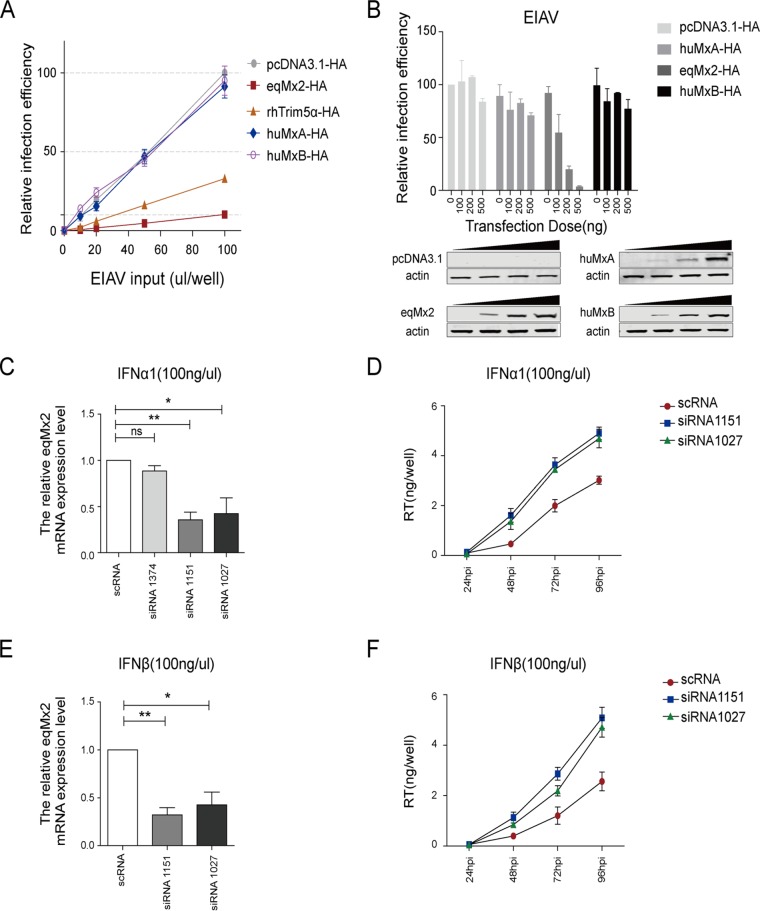
Equine Mx2 restricts infection by both pseudotyped and replication-competent EIAV. (A) HEK293T cells were transfected with 1 μg plasmids expressing either pcDNA3.1, huMxA (negative control), rhTRIM5α (positive control), huMxB, or eqMx2 and challenged with increasing amounts (0, 10, 20, 50, and 100 μl) of a pseudotyped EIAV luciferase reporter virus. Cells were lysed, and the infection efficiency relative to pcDNA3.1-HA was monitored at 24 hpi. Mean relative infection efficiencies with standard deviations from three independent experiments are shown. (B) HEK293T cells were transfected with increasing amounts of huMxA, eqMx2, and huMxB expression plasmids (0, 100, 200, and 500 ng) and inoculated with 2 ng^RT^ pseudotyped EIAV luciferase reporter virus at 24 hpi. Cells were lysed, and luciferase activity in the cell lysates was measured at 24 hpi. The data represent the means ± SE from three independent experiments. Western blotting was used to confirm the expression of certain Mxs. (C) Knockdown of the mRNA expression level of eqMx2. eMDMs were incubated with 100 ng/μl IFN-α1 for 24 h and transfected with either 50 nM eqMx2-specific siRNA (siRNAs 1374, 1151, and 1027) or 50 nM scrambled siRNA control (scRNA). At 24 hpt, eqMx2 mRNA levels were quantified by real-time PCR. This experiment was performed three times, and means ± SE are shown (**, *P* < 0.01; *, *P* < 0.05; ns, no significance). (D) Knockdown of eqMx2 increases EIAV replication in equine macrophages. eMDMs were treated with 100 ng/μl IFN-α1 and transfected with 50 nM siRNA 1151 or 1027 or scRNA for 24 h (siRNA 1374 proved to be ineffective). The cells were then infected with 1 ng^RT^ of DLV36 (a replication-competent EIAV strain). The viral titers in the supernatant were determined at 24, 48, 72, and 96 hpi by measuring the reverse transcriptase (RT) activity. The data represent the means ± SE from three independent experiments. (E and F) Same experiment as in panels C and D, except that the eMDMs were treated with IFN-β and were not transfected with the ineffective siRNA 1374.

An EIAV pseudotyped virus containing a luciferase reporter gene, packaged by EIAV Gag-Pol and the vesicular stomatitis virus glycoprotein (VSV-G) expressed using separate expression vectors, was generated to test the effect of eqMx2 on EIAV replication. rhTRIM5α, which has been reported to block the early life cycle of EIAV (28), was employed as a positive control. HEK293T cells were transfected with either the expression vector for eqMx2, huMxA, huMxB, or rhTRIM5α or an empty vector control. After inoculation with the EIAV pseudotyped virus at 24 hpt, luciferase activity was detected at 24 hpi. The results of this experiment demonstrate that both rhTRIM5α and eqMx2 can significantly reduce EIAV replication (Fig. 4A). However, the huMxB protein could not influence the infection efficiency of EIAV, which is consistent with previously reported research (11, 22). Different levels of eqMx2 and huMxB expression showed that infection with EIAV could be significantly inhibited by eqMx2 but not by huMxB (Fig. 4B).

Having found that the overexpression of the eqMx2 protein is sufficient to prevent the replication of EIAV pseudotyped virus, we wanted to know if endogenous eqMx2 also plays an important role in the type I interferon-induced antiviral state. The basic expression level of eqMx2 was fairly low in eMDMs, so we treated the cells with interferon first. Twenty-four hours later, either three specific short interfering RNAs (siRNAs) (siRNAs 1374, 1151, and 1027) targeting equine Mx2 mRNA or a nontargeting scrambled siRNA (scRNA) was transfected. The antibody from human Mx2 cannot recognize the expression of the equine Mx2 protein, so we tested the mRNA expression level. Levels of expression of eqMx2 mRNA were clearly reduced by interference by siRNA 1151 and siRNA 1027, indicating that the knockdown of eqMx2 is effective (Fig. 4C and E). Another siRNA (siRNA 1374) proved noneffective, and we did not test it further. The cells transfected with either siRNA 1151, siRNA 1027, or control scRNA were then challenged with FDDV_DLV36_ (DLV36) (a replication-competent EIAV strain). The EIAV replication level was monitored by detecting viral reverse transcriptase (RT) in the supernatant at 24, 48, 72, and 96 h postinfection. The results showed that the knockdown of eqMx2 in eMDMs weakens the inhibitory effects of IFN-α1 (Fig. 4D) and IFN-β (Fig. 4F) toward EIAV, which further shows that eqMx2 contributes to type I interferon-induced resistance to EIAV infection. In short, our data show that eqMx2 inhibits the replication of both pseudotyped and replication-competent EIAV.

### The antiviral activity of equine Mx2 protein exhibits substrate selectivity.

**FIG 5:**
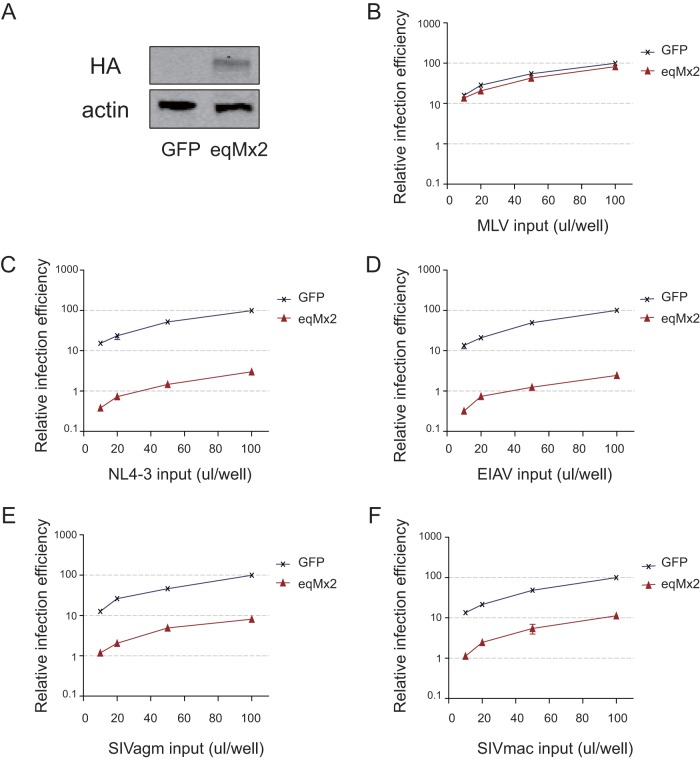
The antiviral activity of eqMx2 exhibits substrate selectivity. A HEK293/eqMx2 cell line (eqMx2) constitutively expressing HA-tagged eqMx2 was constructed. A HEK293/GFP cell line (GFP) was used as the negative control. (A) Immunoblot analysis of HA-tagged eqMx2 protein. Staining for actin served as a loading control. (B to F) Both eqMx2 and GFP cell lines were inoculated with increasing amounts (10, 20, 50, and 100 μl) of one-life-cycle MLV (B), HIV-1 (NL4-3) (C), EIAV (D), SIVagm (E), and SIVmac (F) luciferase reporter viruses. Cells were lysed, and luciferase activity in the cell lysates was measured at 24 hpi. Mean relative infection efficiencies relative to GFP cell lines with standard deviations from three independent experiments are shown.

Results from previous studies suggest that the restriction effect of human MxB is limited to primate lentiviruses, and nonprimate lentiviruses were nonsensitive to suppression by this protein (11, 12). To evaluate the restriction range of eqMx2, a HEK293 cell line (HEK293/eqMx2) constitutively expressing the equine Mx2 protein was developed. The cDNA of eqMx2 was cloned into a pLPCX retroviral vector with three HA tags at the C terminus and cotransfected with murine leukemia virus (MLV) Gag-Pol and VSV-G helper plasmids. A GFP-expressing HEK293 cell line was also made, as a negative control. An HA tag identified by Western blotting confirmed eqMx2 protein expression (Fig. 5A). A range of luciferase-expressing reporter retroviruses, including the gammaretrovirus MLV and the lentiviruses HIV-1_NL4-3_ (NL4-3), EIAV, and simian immunodeficiency virus (SIV) derived from African green monkey (SIVagm) and rhesus macaque (SIVmac), were examined. The results showed decreases in the relative infection efficiencies of NL4-3 and EIAV of more than 90% (Fig. 5C and D) and decreases of nearly 90% for the SIVs (SIVagm and SIVmac) (Fig. 5E and F). However, the equine Mx2 protein has no ability to inhibit the gammaretrovirus MLV (Fig. 5B), a property that it shares with the human MxB protein (11). Taken together, these results confirm that, unlike human MxB, the equine Mx2 protein can suppress not only lentiviruses from primates but also lentiviruses from other mammals, such as EIAV.

### The antiviral function of equine Mx2 requires its amino-terminal domain.

**FIG 6:**
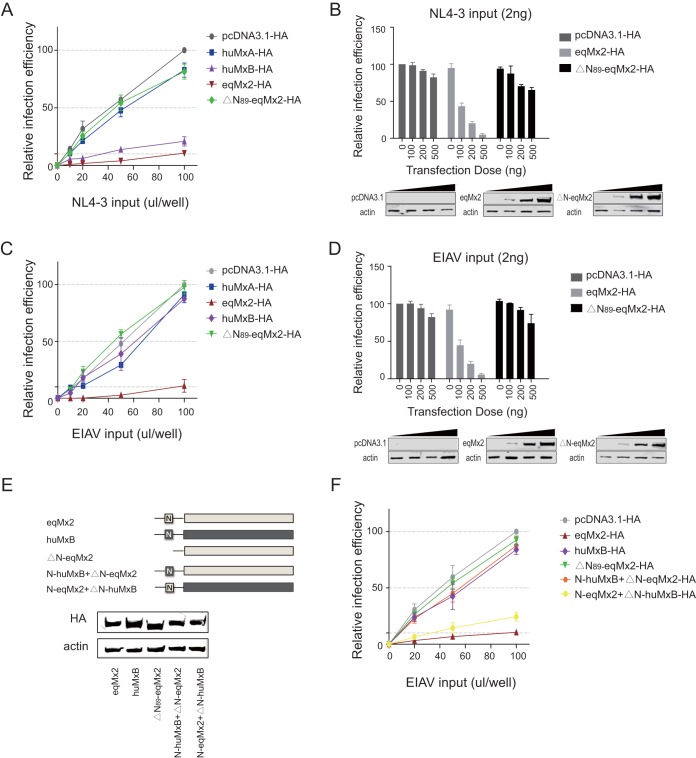
Inhibition of HIV-1 and EIAV by eqMx2 requires the N-terminal domain. (A) Effect of an eqMx2 N-terminal domain deletion (ΔN_89_-eqMx2) on replication of HIV-1. HEK293T cells were transfected with 1 μg plasmid pcDNA3.1 or plasmids expressing huMxA (negative control), huMxB (positive control), eqMx2, or ΔN_89_-eqMx2 and inoculated with increasing amounts (0, 10, 20, 50, and 100 μl) of HIV-1 luciferase reporter virus (NL4-3). Cells were lysed, and the infection efficiency relative to pcDNA3.1-HA was monitored at 24 hpi. Mean relative infection efficiencies with standard deviations from three independent experiments are shown. (B) HEK293T cells were transfected with increasing amounts (0, 100, 200, and 500 ng) of pcDNA3.1-HA or an eqMx2 or ΔN_89_-eqMx2 expression plasmid and inoculated with 2 ng^RT^ NL4-3 at 24 hpi. Cells were lysed, and luciferase activity in the cell lysates was measured at 24 hpi. The data represent the means ± SE from three independent experiments. (C and D) Same experimental procedure as for panels A and B, except that inoculation was performed with the EIAV luciferase reporter virus. Different doses of eqMx2 or ΔN_89_-eqMx2 were identified by Western blotting, and actin was used as a loading control. (E) Schematic representation illustrating the huMxB (dark gray), eqMx2 (light gray), and chimeric eqMx2/huMxB (ΔN_89_-eqMx2, N-huMxB+ΔN-eqMx2, and N-eqMx2+ΔN-huMxB) proteins. Parallel samples as described above were analyzed by Western blotting. (F) HEK293T cells were transfected with 1 μg plasmids from panel E and challenged with increasing amounts (0, 20, 50, and 100 μl) of EIAV luciferase reporter virus. Cells were lysed, and the infection efficiency relative to pcDNA3.1-HA was monitored at 24 hpi. The data represent the means ± SE from three independent experiments.

The N-terminal domain of human MxB has been demonstrated to be vital in the restriction of HIV-1 replication (11, 12, 17, 20, 22–24, 31). We observed that there are great differences between the N-terminal regions of eqMx2 and huMxB (Fig. 2A). To test whether the N terminus of eqMx2 is functional, we deleted 89 residues in the eqMx2 N-terminal domain (ΔN_89_-eqMx2) (Fig. 6E) and examined the antiviral ability of eqMx2 using EIAV and HIV-1 pseudotyped luciferase-expressing reporter viruses. Our results show that when eqMx2 lacks the N-terminal region (ΔN_89_-eqMx2), its antiviral activity is almost totally lost (Fig. 6A and C). The luciferase activities of the single-cycle EIAV and HIV-1 pseudotyped reporter viruses progressively decrease with increasing expression levels of eqMx2 with an intact N terminus, while this restriction effect almost disappears when the N terminus is missing (Fig. 6B and D).

Based on the different restrictive activities of huMxB and eqMx2 toward EIAV, we constructed a set of chimeric huMxB/eqMx2 proteins to further investigate this. As shown in Fig. 6E, the chimera N-huMxB+ΔN-eqMx2 is comprised of the 91 N-terminal amino acid residues from human MxB transferred to a truncated equine Mx2 protein lacking the N-terminal 89 residues, and similarly, the chimera N-eqMx2+ΔN-huMxB is comprised of the N-terminal 89 residues of equine Mx2 fused with a truncated human MxB protein lacking the N-terminal 91 residues. We overexpressed these proteins in HEK293T cells and evaluated the replication level of the EIAV pseudotyped reporter virus. The N-huMxB+ΔN-eqMx2 chimeric protein lost its antiviral activity toward the EIAV pseudotyped virus, which means that the N terminus of huMxB has no effect on EIAV. Meanwhile, although the effect is not as great as for eqMx2 itself, the N-eqMx2+ΔN-huMxB chimeric protein obtained some ability to restrict EIAV (Fig. 6F). In summary, these results showed that the N-terminal domain of equine Mx2 is crucial for the ability of this protein to suppress the replication of EIAV and HIV-1.

### Subcellular localization of equine Mx2.

**FIG 7:**
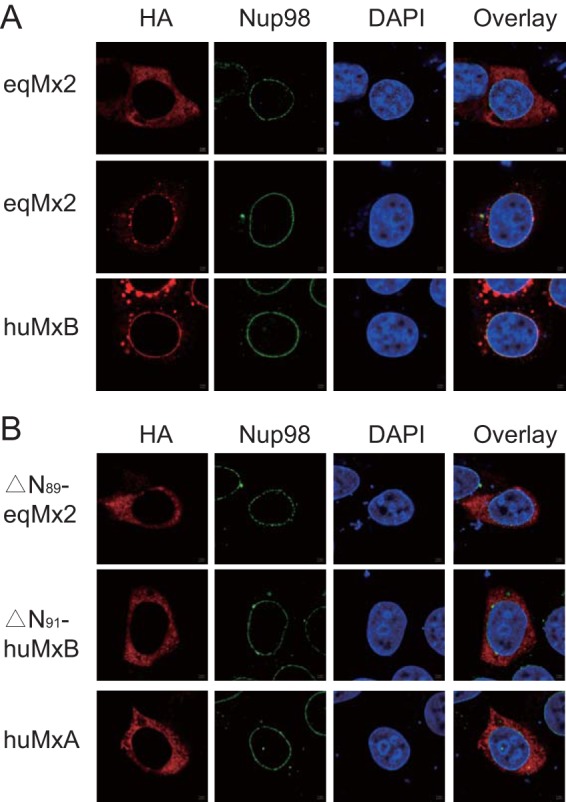
Subcellular localization of equine Mx2. HeLa cells were transfected with plasmids expressing eqMx2 and huMxB (A) or ΔN_89_-eqMx2, △N_91_-huMxB, and huMxA (B), which were tagged with HA at the C terminus. At 24 hpt, the cells were fixed and stained with an anti-HA antibody (TRITC) (red) or anti-Nup98 (FITC) (green), and nuclei were stained with the fluorescent dye DAPI (blue). Two examples of cells expressing eqMx2 are shown in the first and second groups of panel A. This experiment was performed three times, and a representative result is shown.

**FIG 8:**
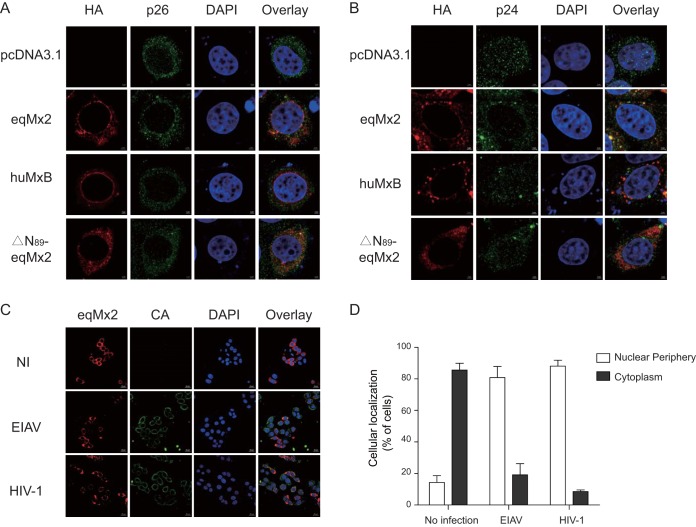
Change in the cellular distribution and capsid binding of equine Mx2 following virus infection. (A and B) Localization of certain Mx2s following EIAV and HIV-1 infection. HA-tagged eqMx2-, huMxB-, or ΔN_89_-eqMx2-expressing plasmids were transfected into HeLa cells, followed by challenge with 20 ng^RT^ EIAV (A) or HIV-1 (B) pseudovirus at 24 hpt. Twenty-four hours later, cells were fixed and stained with an anti-HA antibody (TRITC) (red) or an anti-p26 or -p24 antibody (FITC) (green), nuclei were stained with DAPI (blue), and expression was analyzed using Zeiss confocal microscopy. (C) Representative overview image of the position distribution change of eqMx2. NI, no infection; EIAV, EIAV infection; HIV-1, HIV-1 infection. (D) Analysis of the subcellular localization of eqMx2. This result was determined visually for 200 randomly selected cells using a 40× objective. Mean values with standard deviations from three independent experiments are shown.

Goujon et al. observed that the anti-HIV-1 ability of human MxB was closely correlated with its localization on the nuclear envelope (23). Since equine Mx2 proved to be an effective inhibitor of many lentiviruses, we speculated whether its function was also connected with its subcellular location. Therefore, we used indirect immunofluorescence and confocal microscopy to examine the localization of HA-tagged eqMx2, huMxB, ΔN_89_-eqMx2, ΔN_91_-huMxB, and huMxA in transiently transfected HeLa cell monolayers. The results indicated two distinct patterns of localization for equine Mx2. In 85.7% of the cells, the equine Mx2 protein is predominantly localized throughout the cytoplasm. In the other 14.3% of the cells, the cellular localization is similar to that of human MxB, i.e., mainly accumulating at the nuclear envelope (marked by Nup98) and with very little being found in the cytoplasm (Fig. 7A and 8C and D). In keeping with data from previous studies (23, 31), the deletion of the N-terminal region (ΔN_89_-eqMx2 and ΔN_91_-huMxB) altered the subcellular locations of both human and equine Mx2 proteins, which were dispersed in the cytoplasm with some granules, like the human MxA protein (Fig. 7B). This cytoplasmic localization was connected to a loss of lentivirus suppression function (Fig. 6A and C). Taken together, these results suggest that the localization of Mx2s at the nuclear periphery may contribute to their antilentivirus activity.

### Cell distribution change and capsid binding of eqMx2 following virus infection.

There was a contradiction between the low percentage of nuclear-localized equine Mx2 and its potent antilentivirus function. Therefore, we surmised that the cellular localization of equine Mx2 might be altered following virus infection. To further investigate this, HeLa cells were transfected with eqMx2, huMxB, or N-terminally truncated mutants of equine Mx2 (ΔN_89_-eqMx2). To aid in the detection of the expression of the virus capsid (CA), the cells were challenged with a large amount of EIAV or HIV-1 pseudovirus (at 20 ng reverse transcriptase [RT] activity). Twenty-four hours following EIAV inoculation, the eqMx2 protein, visualized using indirect immunofluorescence, was localized around the cell nucleus in 80.8% of the cells studied (Fig. 8A, C, and D). Twenty-four hours following HIV-1 inoculation, the proportion of cells in which eqMx2 was localized to the nuclear periphery was 88.2% (Fig. 8B to D). However, the cellular locations of huMxB and ΔN_89_-eqMx2 were not changed by lentivirus infection (Fig. 8A and B).

**FIG 9:**
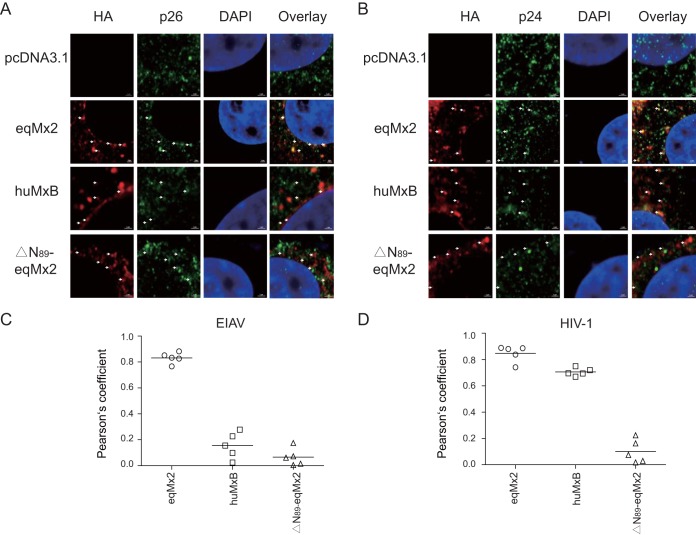
(A) Colocalization of eqMx2 and the EIAV capsid protein. An expanded segment of an optical section of the colocalization between the viral capsid and certain Mx2s is shown. The cells were stained with an anti-HA antibody (TRITC) (red) or an anti-p26 antibody (FITC) (green), and nuclei were stained with DAPI (blue). These cells were examined by confocal microscopy. (B) Same as panel A, except that in this experiment, the cells were challenged with HIV-1 and stained with p24 antibody. This experiment was performed three times, and representative results are shown. (C) Pearson correlation coefficient values for the colocalization of EIAV capsid protein p26 and certain Mx proteins. Symbols signify the Pearson correlation coefficient for the corresponding spots marked by arrows in panel A. (D) Pearson correlation coefficient values for the colocalization of HIV-1 capsid protein p24 and certain Mx proteins. Symbols signify the Pearson correlation coefficient for the corresponding spots marked by arrows in panel B.

Previous studies confirmed that viral CA is a dominant determinant of HIV-1 suppression by human MxB (16, 17, 22). Mutations in CA could help HIV-1 escape recognition by human MxB (11, 12, 31, 32), suggesting that the restrictive function of equine Mx2 may be related to the incoming viral capsid proteins. To investigate potential mechanisms, we particularly stained the samples with antibodies against Mx2s and lentiviral capsids. Ninety-three percent of the cells showed a colocalization of eqMx2 and the EIAV capsid protein p26, and both proteins were found around the cell nucleus (Fig. 8C and 9A and C). However, no colocalization was observed for the p26 protein and huMxB, which could be an explanation for the inability of huMxB to block EIAV infection (Fig. 9A and C). As for HIV-1, both eqMx2 and huMxB showed colocalization with the capsid protein p24, with proportions of 90% and 91.5% of cells examined, respectively (Fig. 9B and D). The N-terminally truncated mutant of eqMx2, however, cannot be colocalized with either the p26 or p24 protein, consistent with its lack of antivirus activity (Fig. 9A to D). Our data demonstrated that the cellular distribution of eqMx2 is altered following lentivirus infection and shows obvious colocalization with the viral capsid, a result that was not observed using an Mx with no antiviral activity.

## DISCUSSION

Innate immunity is the first line of defense against pathogen infection. Mammalian cells encode multiple innate restriction proteins to respond to virus infection (33, 34). Human restriction factors produced in response to HIV-1 infection include APOBEC3 (35), Trim5α (36), tetherin (37), SAMHD1 (38), MOV10 (39), MxB (11–13), SERINC5 (40, 41), and others. Mx genes are key antiviral effectors and are regulated by the interferon signaling pathway (1). Human MxB has been reported to be a potent type I IFN-mediated inhibitor to block HIV-1 infection (11–13). To date, however, the antiviral functions of Mx2s have been observed only in humans and certain other primates. Antiviral activity of nonprimate Mx2 has not been reported. Our data revealed that the mRNA expression of eqMx2 is noticeably upregulated by IFN-α/β. This is in agreement with findings for human MxB (11–13). When we checked other equine cellular restriction factors, the increased upregulation was not so obvious. It remains to be determined whether these genes are stimulated by other types of interferon or require extra time to be upregulated.

Busnadiego et al. demonstrated that neither the ovine nor canine Mx2 protein displayed any antiviral activity (22). Since the expression of eqMx2 could be regulated by type I interferon, we questioned if equine Mx2 has the same antiviral ability as human MxB. Our results demonstrated that the overexpression of eqMx2 decreases infection by HIV-1 and SIVs by nearly 90%. In addition, EIAV was also sensitive to inhibition by eqMx2. Although our laboratory does not have the plasmids to produce other types of lentiviruses, the current data are enough to show that the restriction range of eqMx2 is a little broader than that of huMxB. MLV was insensitive to restriction by eqMx2, and this is consistent with research on huMxB (11, 12) indicating no inhibitory activity of Mx2 proteins toward gammaretroviruses. Gene knockdown experiments provided further evidence. This experiment was performed on equine primary peripheral blood mononuclear cells, so interference by eqMx2 was not very prominent. However, the results showing that EIAV replication was increased in eqMx2 knockdown cells were repeatable. We therefore conclude that the antiviral activity of eqMx2 is not due merely to the pseudotyped virus but also worked on replication-competent virus. These results also demonstrate that the function of eqMx2 is closely connected with the induction of interferon.

It has been reported that the N-terminal region of human MxB is indispensable for HIV-1 restriction (11, 12, 17, 20, 22–24, 31) and has an important role in nuclear localization (21–23, 31) and HIV-1 capsid binding (14, 16, 17, 20, 22, 24). The residues ^11^RRR^13^ are responsible for the ability of huMxB to bind the HIV-1 capsid core during infection and to block HIV-1 infection (20, 24). Our data showed that the deletion of the N terminus of eqMx2 also almost totally abolished its antiviral activity. Construction of the chimeras N-huMxB+ΔN-eqMx2 and N-eqMx2+ΔN-huMxB further supported this conclusion. These findings indicated that Mx2s derived from different species share similar functional domains for lentivirus restriction and that the range of recognition is determined by their N-terminal domains. The specific residues in the amino-terminal domain of eqMx2 that are related to its antiviral ability need to be further evaluated.

The inhibition of HIV-1 by human MxB occurs following the production of proviral cDNAs and prior to the generation of new viral DNAs (11–13). The full-length human MxB gene contains a putative nuclear localization signal on its N-terminal domain (21) and is reported to localize around the cell nucleus (10). As for equine Mx2, our results showed that its N terminus is different from that of huMxB, and it displayed two patterns of subcellular localization. In most uninfected cells, it was found dispersed throughout the cytoplasm. However, following virus infection, its location was altered, and an increased proportion of the protein was found distributed around the nucleus. The colocalization of eqMx2 and lentivirus capsids, as well as the reductions in levels of HIV-1 2-LTR circular DNA, indicated that eqMx2 targets the viral capsid and blocks the nuclear entry of proviral cDNAs, which is in accordance with previous studies showing that huMxB is key for the regulation of the kinetics of nuclear uptake (10) and that the viral CA protein plays an important role in the nuclear import of the viral genome (42–44). The translocation of eqMx2 was closely connected with the incoming viral capsid, and we presumed that the eqMx2 proteins may change the role from some basic cell function and become “gatekeepers,” guarding the nucleus against importing the proviral cDNA. For the convenience of observing the expression of virus capsid, we added a massive dose of virus that is much higher than the dose of Mx2s. The restriction effect of antiviral protein is closely connected with both doses. With careful observation of the colocalization images in Fig. 8, some p26 or p24 protein is detectable in the nucleus. However, this does not influence the final conclusion. Previous studies suggested that there are numerous host cellular proteins, including CPSF6, TNPO3, Nup358 (also known as RanBP2), and Nup153, that interact with CA and are associated with trafficking from the cytoplasm to the nucleus (44, 45). Therefore, specific host molecules involved in lentivirus suppression through eqMx2 remain to be determined.

In conclusion, our study reveals that the eqMx2 protein is a nonprimate interferon-inducible antiviral effector restricting the replication of many lentiviruses by binding and blocking the nuclear entry of viral capsid. Its special N-terminal region determines the capsid binding capacity as well as the range of antiviral activity. These findings expand our understanding of the Mx2 proteins, although further studies investigating the intermolecular interactions of the Mx-mediated antiviral process are needed.

## MATERIALS AND METHODS

### Plasmids.

eqMx2 cDNA was cloned by RT-PCR from total RNA derived from equine monocyte-derived macrophages (eMDMs), induced by equine IFN-α1 (100 ng/μl) (Kingfisher Biotech) for 24 h. The primers were designed according to the genomic sequence of the Equus caballus MX dynamin-like GTPase 2 (Mx2) gene (GenBank accession number XM_005606159.2). The amplified fragments were cloned into the pcDNA3.1-HA vector, which is a pcDNA3.1(+) vector (Invitrogen) with 2×HA tags at the C terminus. The human MxA and MxB genes were purchased from Summus Co. (China) and also cloned into the pcDNA3.1-HA vector. The N termini of the truncated forms of equine Mx2 (ΔN_89_-eqMx2) and human MxB (ΔN_91_-huMxB) were deleted by PCR based on eqMx2 and human MxB cDNAs. The cDNAs encoding the chimeras N-huMxB+ΔN-eqMx2 and N-eqMx2+ΔN-huMxB were obtained by overlapping PCRs and cloned into pcDNA3.1-HA vectors. All constructed mutants were confirmed by sequencing. Rhesus macaque TRIM5α (rhTRIM5α) was synthesized as previously described (30).

### Cells and virus stocks.

Human embryonic kidney HEK293T cells and HeLa cells were cultured at 37°C in a 5% CO_2_ incubator in Dulbecco's modified Eagle's medium (HyClone) supplemented with 10% fetal bovine serum (Sigma) and 1% penicillin-streptomycin. eMDMs were prepared from equine peripheral blood mononuclear cells (PBMCs) as described previously (46) and maintained in RPMI 1640 (HyClone) supplemented with 30% horse serum (HyClone) and 30% fetal bovine serum (HyClone). The replication-competent EIAV strain FDDV_DLV36_ (DLV36) was stocked in this laboratory and was titrated using an RT assay kit (Roche) according to the manufacturer's instructions. The HIV GFP reporter virus was taken from laboratory stocks (30).

### Measurement of gene expression by qPCR.

eMDMs were seeded into 24-well plates and incubated at 37°C for 48 h to obtain confluent monolayers. Cells were treated for 24 h with 100 ng/μl equine IFN-α1 (Kingfisher Biotech) or IFN-β (Abbexa Ltd.). Total cellular RNA was extracted using the RNeasy plus minikit (Qiagen) and reverse transcribed into cDNA using a reverse transcription kit (TaKaRa) according to the manufacturer's instructions. The cDNA preparations were subjected to real-time quantitative PCR (qPCR) analysis using the SYBR green PCR mixture. Real-time PCR was performed using the eqMx2-specific primers 5′-TAGCTGGGAATGGAGTTG-3′ (forward) and 5′-CACCAGGTTGATCGTCTC-3′ (reverse). The cDNA of β-actin was prepared as a housekeeping control and quantified using qPCR. Relative mRNA expression levels were determined using the 2*^−ΔΔCT^ method*.

### Construction of luciferase-expressing reporter virus and its infection.

A luciferase-expressing EIAV pseudotyped reporter virus that contained a luciferase reporter gene was constructed with EIAV Gag-Pol and vesicular stomatitis virus glycoprotein (VSV-G) (47). HEK293T cells were cotransfected with 9 μg of pONY8.1-LUC, 9 μg of pEIAV-GagPol, and 3 μg of VSV-G. The virus was collected at 48 h posttransfection (hpt) and used to infect certain protein-expressing HEK293T cells seeded into 24- or 48-well plates. These cells were washed and subjected to luciferase analysis at 24 h postinfection (hpi). The VSV-G-pseudotyped luciferase-expressing HIV reporter virus (NL4-3) was constructed in the same way, with the transfection dose adjusted to 16 μg pNL4-3_lucΔVif,ΔEnv and 4 μg VSV-G. The SIV reporter virus was also produced in the same way. The HIV-1 GFP reporter virus was taken from laboratory stocks (30).

### qPCR analysis of HIV-1 2-LTR DNA.

HEK293T cells were transfected with either 1 μg empty vector or eqMx2- or huMxB-expressing plasmids. The cells were then challenged with HIV-1_NL4-3_ luciferase reporter virus at 24 hpt. Total cellular DNA was extracted using the DNeasy tissue kit (Qiagen) at 24 hpi, and 500 ng of each sample was subjected to qPCR analysis. HIV-1 2-LTR circular DNA was amplified with the primer pair 2-LTR circle-F (5′-CCCTCAGACCCTTTTAGTCAGTG-3′) and 2-LTR circle-R (5′-TGGTGTGTAGTTCTGCCAATCA-3′) and with a 2-LTR circle probe (5′-FAM [6-carboxyfluorescein]-TGTGGATCTACCACACACAAGGCTACTTCC-TAMRA [6-carboxytetramethylrhodamine]-3′) (48). GAPDH was used as a housekeeping control to normalize the number of living cells.

### Knockdown of eqMx2 expression in eMDMs by siRNA.

Three eqMx2-specific short interfering RNAs (siRNAs) and a scramble siRNA negative control (scRNA) were synthesized by Sigma. eMDMs were seeded into 48-well plates and cultivated for 2 days. These cells were treated with 100 ng/μl equine IFN-α1 (Kingfisher Biotech) or IFN-β (Abbexa Ltd.) for 24 h before being transfected with either eqMx2-specific or negative-control siRNA, which was diluted to 50 nM in serum-free medium. The knockdown efficiency of eqMx2 mRNA was verified by real-time PCR.

### Construction of the eqMx2-expressing HEK293 cell line.

The cDNA of eqMx2 was cloned into a pLPCX retroviral vector with 3×HA tags at the C terminus. The resulting plasmid was sequenced and then cotransfected with the MLV Gag-Pol and VSV-G helper plasmids for 48 h to generate pseudotyped viruses. HEK293 cells were infected with the recombinant virus and screened using puromycin. Through limiting dilution and proliferation, a cell line that consistently expressed eqMx2 was purified and finally identified using Western blotting.

### Transfection and Western blotting.

Cells were transiently transfected with the indicated plasmids using the calcium phosphate method or with PolyJet DNA transfection reagent (SignaGen Laboratories), according to the manufacturer's instructions. At 48 h posttransfection, the cells were lysed in buffer containing 150 mM Tris-HCl (pH 7.6), 50 mM NaCl, 5 mM EDTA, and 1% Triton X-100. The proteins in the cell lysates were separated on 4 to 12% gels by SDS-PAGE. The separated proteins were then transferred onto nitrocellulose membranes and blocked with 5% bovine serum albumin in Tris-buffered saline (TBS) for 2 h at room temperature. Membranes were incubated for 2 h with the appropriate primary antibodies, which included a mouse monoclonal anti-HA antibody (Sigma) and a mouse antiactin antibody (Sigma). The membranes were washed three times in TBS buffer with Tween 20 (TBST) for 10 min each time and then incubated with the relevant secondary antibody for 1 h at room temperature. Specific proteins were detected and quantified using the Odyssey system (Li-Cor).

### Immunostaining and immunofluorescence microscopy.

HeLa cells were transiently transfected with 1 μg eqMx2 and huMxB plasmids alone or infected with VSV-G-packed EIAV pseudotyped viruses 24 h later. The cells were then fixed with 4% paraformaldehyde for 30 min and permeabilized in 0.1% Triton X-100 for 15 min. The cells were incubated with anti-HA or -p26 antibodies (made in-house) (49) or anti-p24 antibodies (provided by Yongtang Zheng) (50), followed by staining using secondary antibodies conjugated with fluorescein isothiocyanate (FITC) or tetramethyl rhodamine isocyanate (TRITC) (Sigma). Nuclei were labeled with 4′,6-diamidino-2-phenylindole (DAPI) (Beyotime, China). Images were acquired using a confocal microscope (LSM 880; Zeiss, Germany). Pearson correlation coefficient values for certain Mx2s and the viral capsid protein were analyzed for 5 suspected colocalization points marked by white arrows in Fig. 9A and B using ZEN software (51).

### Accession number(s).

Sequences were deposited in GenBank under accession number MH428802.
